# *Early* Detection of *Widespread* Progressive Brain Injury after Cardiac Arrest: A Single Case DTI and Post-Mortem Histology Study

**DOI:** 10.1371/journal.pone.0092103

**Published:** 2014-03-14

**Authors:** Jan S. Gerdes, Ernst U. Walther, Suad Jaganjac, Maria Makrigeorgi-Butera, Sven G. Meuth, Michael Deppe

**Affiliations:** 1 Department of Neurology, Schön Klinik Hamburg Eilbek, Hamburg, Germany; 2 Department of Radiology, Schön Klinik Hamburg Eilbek, Hamburg, Germany; 3 MVZ Hanse Histologikum GmbH, Hamburg, Germany; 4 Department of Neurology, University of Münster, Münster, Germany; Julius-Maximilians-Universität Würzburg, Germany

## Abstract

**Objective:**

We tested the hypothesis in sense of a proof of principle that white matter (WM) degeneration after cardiopulmonary arrest (CPA) can be assessed much earlier by diffusion tensor imaging (DTI) than by conventional MRI.

**Methods:**

We performed DTI and T2-weighted FLAIR imaging over four serial acquisitions of a 76-year-old man with unresponsive wakefulness syndrome at day 41, 75, 173 and 284 after CPA. DTI was also performed in ten healthy control subjects. Fractional anisotropy (FA) derived from DTI was assessed in eleven regions of interest within the cerebral white matter (WM) and compared with post-mortem neuropathological findings.

**Results:**

In contrast to conventional FLAIR images that revealed only circumscribed WM damage, the *first* DTI demonstrated significant reduction of FA across the whole WM. The following FLAIR images (MRI 2-4) revealed increasing atrophy and leukoaraiosis paralleled by clinical deterioration with reduction of wakefulness and intractable seizures. Neuropathological findings confirmed the widespread and marked brain injury following CPA.

**Conclusion:**

DTI may help to evaluate microstructural brain damage following CPA and may have predictive value for further evolution of cerebral degeneration in the chronic phase after CPA.

## Introduction

Hypoxic-ischemic encephalopathy (HIE) following cardiopulmonary arrest (CPA) is an important medical issue with challenging ethical implications, since survival after cardiopulmonary resuscitation has become more frequent. Brain injury caused by global cerebral ischemia often results in serious clinical conditions such as coma or unresponsive wakefulness syndrome. Clinical improvement or deterioration in the chronic course of HIE might be observed bedside, however, the neural changes that potentially underlie these clinical changes are poorly understood. White matter (WM) injury is known to occur acute [1], subacute [2] or in a delayed fashion [3] after global cerebral ischemia or anoxia, but little is known about the chronic evolution of WM integrity in patients with HIE. Here we present progressive WM degeneration in a patient with HIE following CPA using longitudinal diffusion tensor imaging (DTI) and its histopathological correlate was assessed *post mortem*.

## Materials and Methods

### Patient and control subjects

A 76-year-old man had a CPA and was resuscitated after an estimated time period of 4–8 min. In hospital, the cause of the CPA could not be detected by computer tomography of the thorax or by angiography of the coronary arteries. The electrocardiogram showed a prolongation of the QT interval. Here “QT” refers to the interval between the start of the Q wave and the end of the T wave in the heart's electrical cycle. Three weeks before CPA, a new therapy with the anti-arrhythmic sodium-channel blocker Flecainide had been started. We assumed the CPA had most likely occurred due to a long QT syndrome after starting Flecainide therapy. The patient was treated with mild hypothermia (34.5°C) for 24 h. Serum neuron-specific enolase (NSE) was elevated to 104.8 μg/L 48 h after CPA (in patients not treated with hypothermia, NSE increases>33 μg/L are suggestive for a poor outcome [4]). Cerebral computer tomography three days after CPA indicated signs of global brain damage. The EEG showed epileptic activity with generalised spike- and polyspike-wave complexes. Clinically, myoclonic twitches had been observed. Therefore, a treatment with valproate (3 g/d) was started. After seventeen days of acute treatment, the patient had been moved to our rehabilitation clinic. On examination, motor responses after painful stimuli were elicited and pupillary and corneal reflexes were present. Both plantar responses were extensor. The patient did not show any clinical improvement over the time course of ten months, but had continuous myoclonic seizures. EEG recordings demonstrated continuous epileptic discharges although antiepileptic drug therapy was gradually increased with high dosages of valproate (5,4 g/d) in combination with levetiracetam (4 g/d), topiramate 200 mg/d and midazolam at various IV infusion rates. The patient died after ten months of best supportive treatment. The present study was conducted after approval of the local ethics committee (Ethik-Kommission Ärztekammer Hamburg). We also studied a group of 10 neurologically healthy controls (median 60.5 years, min 43, max 78). Written informed consent was obtained from all controls and the legal guardian of the patient.

### Magnetic resonance imaging

The patient was admitted for MRI including T1-weighted, T2-weighted, FLAIR, and diffusion tensor imaging (DTI) at four times points (day 41, 75, 173 and 284 after CPA) using a Symphony 1.5 T scanner (Siemens, Erlangen, Germany). For DTI we used single shot echo planar imaging (EPI) with 6 diffusion directions [b-factors 0 and 1000 s/mm^2^, TR = 9.8 s/TE = 95 ms, acquisition matrix: 128×128, voxel size: 1.8×1.8×3.6 mm^3^, two averages]. All EPI images have been smoothed by using a Gaussian kernel of 2 mm×2 mm×4 mm FWHM. An experienced neuroradiologist evaluated findings on conventional MRI. DTI processing was performed by using the “Münster Neuroimaging Evaluation System (EVAL)” [5]–[7]. The employed EVAL-DTI processing pipeline incorporated multi-contrast image registration and correction for eddy currents [7], [8]. Registered fractional anisotropy (FA) images corresponded to the MNI coordinate space. For quantitative comparisons FA was averaged in various regions of interest (ROI), for details see Table 1. All ROIs were created automatically by the EVAL pipeline on the output images from the registration toolbox for the patient and controls, as previously described [5], [9]. The patient's mean FA values of the ROIs were compared longitudinally and with the control group by inferential statistics (*t*-tests, corrected for multiple comparisons).

**Table 1:** The control subjects' and the patient's mean FA values of different ROIs as calculated from DTI. Significance levels (* p<0.05, ** p<0.001, *** p<0.0001).

ROI FA values	Controls		Patient							
	DTI		DTI-1		DTI-2		DTI-3		DTI-4	
	Mean	SD	Mean	% reduction relative to controls	Mean	% change relative to DTI-1	Mean	% change relative to DTI-2	Mean	% change relative to DTI-3
FA whole WM	0.355	0.020	0.223	−37 (**)	0.203	−9	0.188	−7	0.171	−9
FA occipital lobe	0.309	0.020	0.143	−54 (***)	0.122	−14	0.126	3	0.121	−4
FA parietal lobe	0.340	0.021	0.166	−51 (***)	0.148	−11	0.142	−4	0.136	−4
FA frontal lobe	0.346	0.025	0.203	−41 (**)	0.177	−13	0.163	−8	0.139	−15
FA corpus callosum	0.445	0.036	0.247	−45 (**)	0.216	−12	0.195	−10	0.175	−10
FA corona radiata	0.357	0.023	0.199	−44 (**)	0.175	−12	0.157	−10	0.141	−10
FA corticospinal tract	0.389	0.022	0.263	−32 (**)	0.242	−8	0.218	−10	0.202	−7
FA internal capsule	0.420	0.024	0.325	−23 (*)	0.303	−7	0.278	−8	0.260	−6
FA brain stem	0.395	0.020	0.337	−15 (*)	0.324	−4	0.305	−6	0.284	−7
FA temporal lobe left	0.351	0.018	0.214	−39 (***)	0.204	−4	0.189	−7	0.181	−4
FA temporal lobe right	0.348	0.021	0.226	−35 (**)	0.209	−7	0.202	−4	0.196	−3

### Neuropathology

Neuropathology was performed to correlate histopathological hallmarks with MRI findings. The patient's brain was fixed in 4% buffered formalin for four weeks. Coronal sections were made at 0.5 to 1.0 cm intervals. Microscopic sections were taken from cortex and subcortex of frontal, parietal, temporal and occipital lobes. Sections were also taken from the cerebellum, brainstem, thalamus and basal ganglia. These sections were embedded in paraffin for histological examination; 8 μm thick sections were cut and stained with hematoxylin-eosin (HE), van Gieson or with Klüver-Barrera stain. Immunhistochemistry was performed to detect astroglia cells (antibody: anti-GFAP, 1,39 μg/ml, Roche, Basel, Switzerland) and microglia cells/macrophages (antibody: anti-CD68, 0,4 μg/ml, Roche, Basel, Switzerland).

## Results

### DTI

Compared to the group of healthy controls (no significant age difference, *t* = 1.64, *p* = 0.135), the FA of the patient was significantly reduced in every ROI (Table 1). Comparing the patient's FA values of the ROIs derived from DTI-1 with the corresponding FA values of controls, FA was mostly reduced in the occipital (−54%, *p*<0.0001) and parietal lobe (−51%, *p*<0.0001; Table 1).

Relative to the other lobes, the temporal lobe was less affected. From DTI-1 to DTI-4, FA decreased in every ROI beside the putamen. However, despite that the parietal and occipital regions showed the largest FA reduction at DTI-1, these regions showed less FA reduction in the consecutive DTI measures than other ROIs.

### Conventional structural MRI

**Figure 1:**
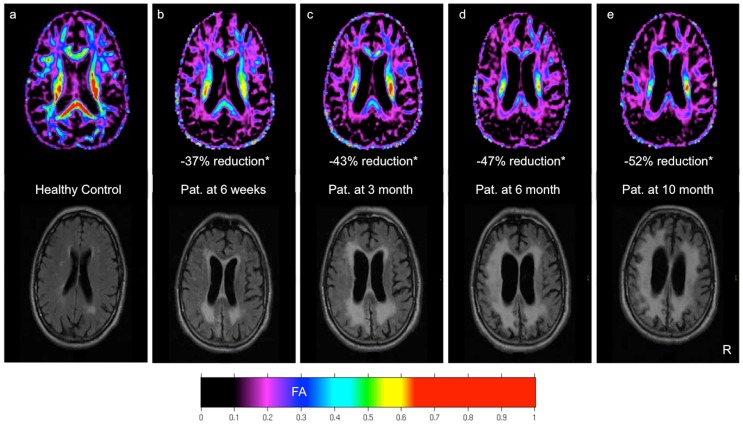
Color-coded FA map and FLAIR MRI of a 78 y old healthy control (a). DTI and MRI of the 76(b–e). * = Reduction of mean FA of the whole white matter (WWM) relative to the mean WWM FA of ten age-matched healthy control subjects.

At MRI-1 the patient showed brain atrophy along with circumscribed leukoaraiosis mainly in parieto-occipital regions. In contrast to the FA images, the FLAIR and T1/T2-weighted images showed at the first MRI scan date wide areas of apparently unaffected WM (Fig. 1). The following FLAIR images revealed increasing atrophy and leukoaraiosis. MRI-4 showed widespread leukoaraiosis extended to the whole WM.

### Neuropathology

**Figure 2:**
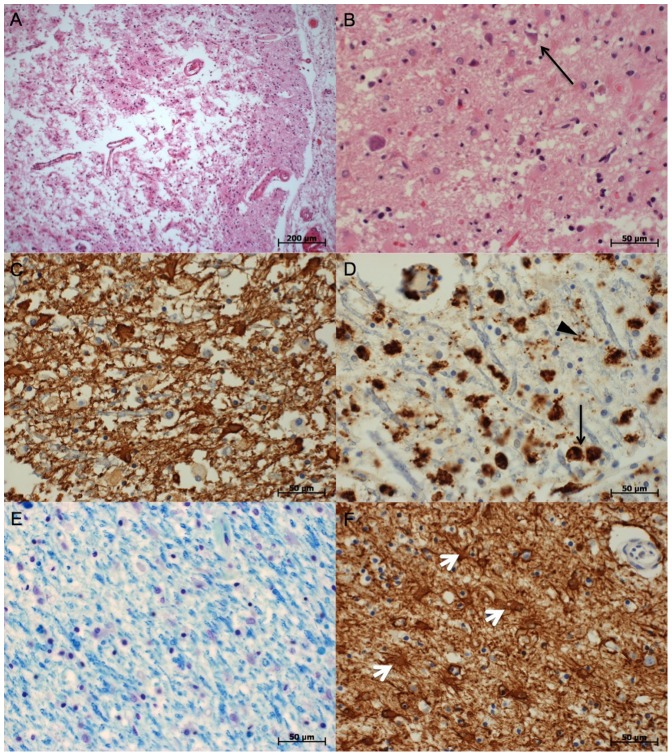
Examples of histopathologic findings. A: Pannecrosis, parietal lobe. HE stain. B: Neuronal necrosis (arrow), frontal lobe. HE stain. C: glial scarring of the white matter. GFAP stain. D: Inflammation of the white matter (black arrows: macrophages, black arrow head: microglia). CD68 stain. E: Patchy loss of myelin structure in the occipital lobe. Klüver-Barrera stain. F: Astrogliosis of the putamen (white arrow heads: astrozytes). GFAP stain.

Neuronal necrosis (Fig. 2B) was multifocal, partly with transition to total necrosis including non-neural tissue and vessels (Fig. 2A). In these areas, reduction of neuronal structure, macrophage activity and reactive astrogliosis was visible. Signs of WM destruction (Fig. 2E), macrophage activity (Fig. 2D) and astrogliosis (Fig. 2C) were found in the corpus callosum and bilaterally in the descending/ascending neural tracts in the frontal, parietal and occipital lobes and to a smaller extent also in the temporal lobes, findings that corresponded well to the FA changes. Further neuronal necrosis was found in the hippocampus, basal ganglia and thalamus.

## Discussion

The present study demonstrates that WM degeneration after cardiopulmonary arrest can be assessed much earlier by DTI than by conventional structural MRI. While the first DTI demonstrated significant FA reduction in the WM globally, the first conventional MRI did not show the full extent of WM damage. The decline of neural structure seems to have steadily continued until death given the longitudinally measured FA decreases. A singular causative event, i.e. global ischemia for 4–8 minutes, unexpectedly resulted in continuous neural degeneration without cessation or restoration.

Delayed neuronal cell death has been described in cases of HIE, especially after prolonged hypoxia [3], the underlying physiopathology of which has not been sufficiently explained to date. Susceptibility of neurons and oligodendrocytes to glutamate-induced excitotoxicity following ischemia resulting in delayed apoptosis and consecutively in demyelination of axons could be shown [10]. These processes are accompanied by mitochondrial dysfunction, formation of free radicals, pH-decrease, formation of focal and general edema and activation of ion channels as demonstrated in both animal models of cerebral ischemia and autoimmune inflammation [11]–[14]. Inflammatory responses following ischemic brain injury could also contribute to the delayed progression of the brain injury [15]. Both, loss of myelin and inflammatory responses with macrophage activity were seen in the histologic sections of the patient *post mortem* (Fig. 2). A third possible cause for the progressive neurodegeneration could be that HIE induced epileptic seizures. Seizures, status epilepticus or myoclonic status epilepticus are typical sequelae following CPA and could be a concomitant cause of brain injury [16]. Our patient revealed clinical and electrophysiological signs for epileptic activity until his death, despite intensive antiepileptic drug therapy.

The comparison of the whole brain FA map of DTI-1 with a FA map of a healthy control suggests that the *complete* WM was already altered at the first MRI (Fig. 1). This early seen alteration had no equal correlate in conventional MRI.

## Conclusion

The present findings motivate future longitudinal studies with larger sample-sizes to investigate the relation between microstructural and clinical developments in patients with HIE after CPA. One aim should be the assessment of FA thresholds that may be indicative for adverse or beneficial outcomes of patients with HIE, since no conventional imaging technique could reliably predict neurological outcome after CPA until today [17].
